# Whole-exome sequencing identifies a novel *de novo* mutation in *DYNC1H1* in epileptic encephalopathies

**DOI:** 10.1038/s41598-017-00208-6

**Published:** 2017-03-21

**Authors:** Zhongdong Lin, Zhenwei Liu, Xiucui Li, Feng Li, Ying Hu, Bingyu Chen, Zhen Wang, Yong Liu

**Affiliations:** 1 Institute of Neurobiology, Xi’an Jiaotong University Health Science Center, Xian, China; 2 Department of Pediatric Neurology, The Second Affiliated Hospital and Yuying Children’s Hospital, Wenzhou Medical University, Wenzhou, China; 3 Institute of Genomic Medicine, Wenzhou Medical University, Wenzhou, China; 4 Research Center of Blood Transfusion Medicine, Key Laboratory of Laboratory Medicine, Ministry of Education, Zhejiang Provincial People’s Hospital, Hangzhou, China

## Abstract

Epileptic encephalopathies (EE) are a group of severe childhood epilepsy disorders characterized by intractable seizures, cognitive impairment and neurological deficits. Recent whole-exome sequencing (WES) studies have implicated significant contribution of *de novo* mutations to EE. In this study, we utilized WES for identifying causal *de novo* mutations in 4 parent-offspring trios affected by West syndrome. As a result, we found two deleterious *de novo* mutations in *DYNC1H1* and *RTP1* in two trios. Expression profile analysis showed that *DYNC1H1* and *RTP1* are expressed in almost all brain regions and developmental stages. Interestingly, co-expression and genetic interaction network analyses suggested that *DYNC1H1* and *RTP1* are tightly associated with known epilepsy genes. Furthermore, we observed that the *de novo* mutations of *DYNC1H1* were identified in several different neuropsychiatric disorders including EE, autism spectrum disorders and intellectual disabilities by previous studies, and these mutations primarily occurred in the functional domain of the protein. Taken together, these results demonstrate *DYNC1H1* as a strong candidate and *RTP1* as a potential candidate on the onset of EE. In addition, this work also proves WES as a powerful tool for the molecular genetic dissection of children affected by sporadic EE.

## Introduction

Epileptic encephalopathies (EE) are typically defined as a devastating group of severe childhood epilepsy disorders characterized by early onset of seizures associated with ongoing epileptic activity^1^. West syndrome (MIM 308350) is one of the most common form of infantile epileptic encephalopathy, characterized by tonic spasms with clustering, arrest of psychomotor development, and hypsarrhythmia in electroencephalography (EEG)^1, 2^. Approximately 17% of all cases can evolve into Lennox-Gastaut syndrome (LGS) which is characterized by polymorphic intractable seizures and paroxysms of fast activity in EEG^3^.

In the past few years, several genes, such as *ARX* ^4^, *CDKL5* ^5^ and *SPTAN1* ^6^, have been implicated in West syndrome based on a candidate gene sequencing approach^7, 8^. Furthermore, several recent exome-sequencing studies have shown that West syndrome probands exhibit an excess of loss of function *de novo* mutations, which plays an important role in West syndrome^9^. Indeed, in a study in which exome sequencing was performed in 264 patient-parent trios with EE, *de novo* mutations were recurrently observed in probands within known causative genes for West syndrome, such as *STXBP1* (n = 4) and *CDKL*5 (n = 2)^9^. In other recent studies, *de novo* mutations in *GRIN2B* ^10^, *GNAO1* ^11^, *KCNT1* ^12^ and *SPTAN1* ^13^ found in probands have been recognized as being associated with West syndrome. These findings showed that West syndrome is a genetically heterogeneous condition, and *de novo* mutations play a significant role in the onset of West syndrome.

Recent advances in the whole-exome sequencing (WES) approach have offered a cost-effective method for investigating single-nucleotide variants (SNVs) across all gene-coding regions in the genome. WES has been successfully applied to identify *de novo* mutations associated with neurodevelopmental disorders for autism spectrum disorders (ASDs)^14, 15^, intellectual disabilities (IDs)^16, 17^, schizophrenia (SCZ)^18, 19^ and EE^20, 21^. To obtain insight into the characterization of *de novo* mutations in West syndrome, we conducted WES of 4 unrelated Chinese parent-offspring trios affected by West syndrome. The results revealed two novel *de novo* mutations in *DYNC1H1* and *RTP1* which were predicted to be deleterious based on the concordance of generic damage prediction tools. Furthermore, expression, co-expression and genetic interaction network analyses provided supporting evidences for the role of *DYNC1H1* and *RTP1* in EE. In particular, the *de novo* mutations in *DYNC1H1* were found to be shared among different neuropsychiatric disorders of EE, ASD and ID, which further implicated *DYNC1H1* in the onset of sporadic neuropsychiatric disorders.

## Results

### Detection of *de novo* mutations

WES of 4 EE trios with West syndrome (one affected child and both unaffected parents) was performed. After the removal of sequencing adapters and trimming of low-quality bases, approximately 2.12~4.72 Gb of cleaned sequencing data were obtained for each sample (Supplemental Table 1). More than 99.42% of the sequencing reads were aligned to the human reference genome (hg19), with 56.38% effective reads from target regions being obtained after the removal of PCR duplications. The average sequencing depth for each sample was 70.25-fold, with more than 86.00% of target regions having at least a 10-fold coverage. As a result, 21,756 SNVs or InDels were identified and 3 *de novo* SNVs in coding regions were identified and validated in the 4 trios. We also attempted to identify rare (new or with allele frequency <0.001 based on Exome Aggregation Consortium (ExAC)^22^, 1000 Genomes^23^ and the NHLBI Exome Sequencing Project (ESP)^9^) inherited mutations in known or related epilepsy genes, but no rare inherited mutations were detected for the 4 trios.

### Analysis of the association of *de novo* gene mutations with EE

Recent WES studies based on parent-offspring trios are increasingly demonstrating that *de novo* mutations play a prominent role in a substantial number of EE, although the extent of this contribution is not yet known^24^. In the present study, we observed that two of the three missense *de novo* mutations were clearly predicted to be functionally deleterious based on four mutation effect prediction tools (SIFT, VEST3, LRT and SiPhy) (Table 1).

**Table 1:** Summary of DNMs detected by trios-based WES of EE.

Trio	Chr	Position(hg19)	Gene	Mutation	Transcript	Protein change	SIFT	VEST3	LRT	SiPhy
E1	chr6	158318012	*SNX9*	missense	NM_016224	p.Q152K	tolerable	damaging	neutral	conserved
E2	chr3	186917389	*RTP1*	missense	NM_153708	p.I108N	damaging	damaging	deleterious	conserved
E3	chr14	102499496	*DYNC1H1*	missense	NM_001376	p.M3392V	damaging	damaging	deleterious	conserved

**Figure 1:**
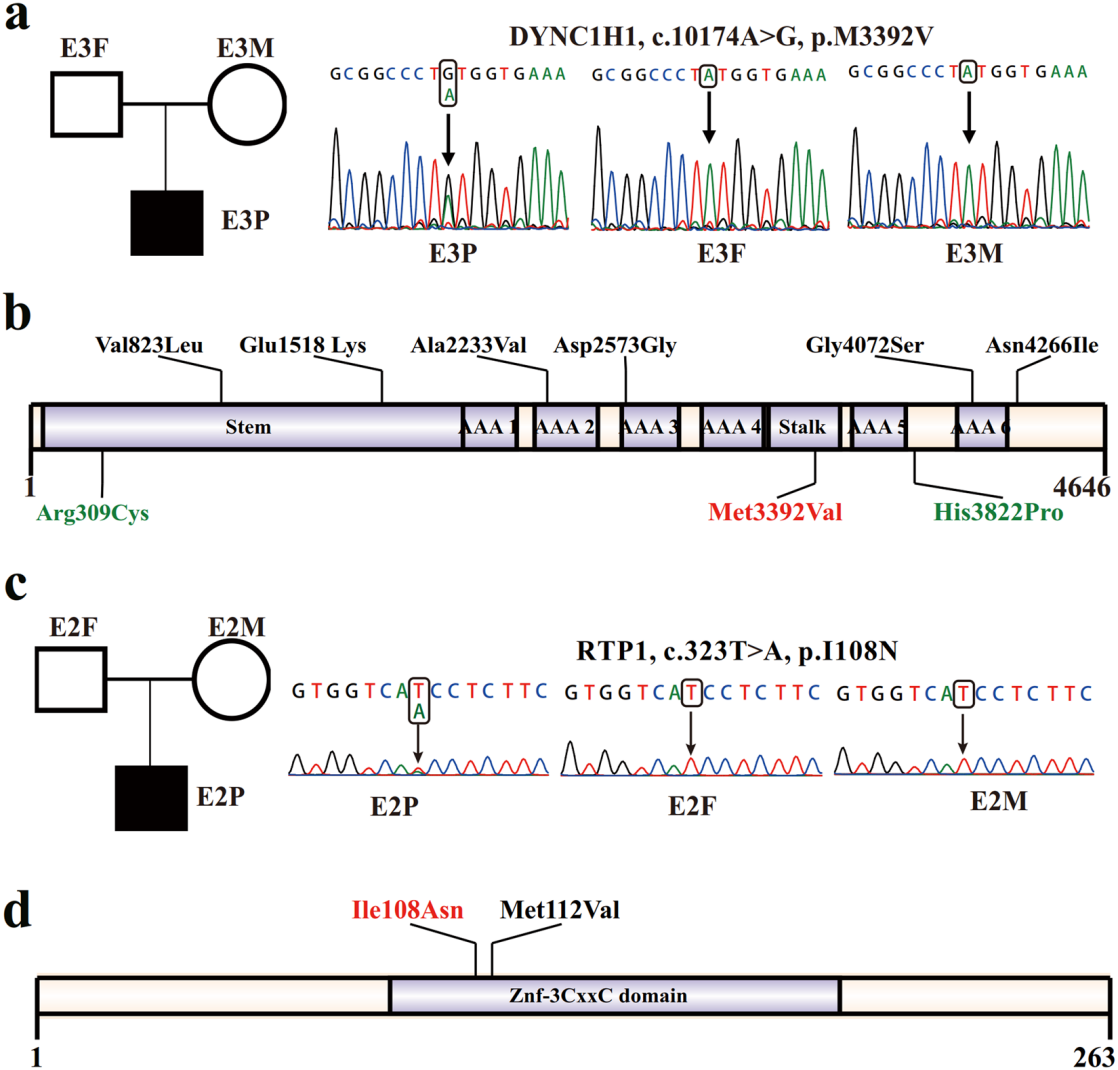
*De novo* mutations identified in *DYNC1H1* and *RTP1*. (**a**) The *de novo* mutation of *DYNC1H1* was confirmed in trio E3 using Sanger sequencing. (**b**) Schematic representation of the DYNC1H1 protein and *de novo* mutations of *DYNC1H1* identified in EE, ASD and ID. Protein changes are shown in black for ASD, green for ID and red for EE. (**c**) The *de novo* mutation of *RTP1* was confirmed in trio E2 using Sanger sequencing. (**d**) Schematic representation of the RTP1 protein and *de novo* mutations of *RTP1* identified in EE. Protein change identified in the present study is shown in red, and those identified in the previous study are shown in black.

One novel *de novo* mutation was detected in *DYNC1H1* in E3P proband. This mutation was nonsynonymous (c.10174A > G) and caused a methionine (Met) to valine (Val) substitution at amino acid 3392 (p.M3392V) in the conserved MT domain (microtubule-binding stalk of dynein motor) (Fig. 1a,b and Table 1). This mutation was predicted as deleterious by the four types of prediction tools employed in this study (Table 1). Furthermore, *DYNC1H1* might be considered as an intolerant gene based on residual variation intolerance score (RVIS)^25^, which was developed to estimate functional variants deviations based on the ESP6500 dataset and Z score for missense variants from ExAC^22^ (Supplemental Table 2).

We also validated a novel missense mutation of T to A substitution (c.323T > A) in *RTP1* (MIM 609137) in E2P proband (Fig. 1c and Table 1). The single nucleotide change on *RTP1* produced a nonsynonymous substitution (p.I108N) (Fig. 1d). Similarly, this mutation was considered to be deleterious according to prediction software tools (Table 1). Although *RTP1* tends to be not evolutionarily constrained based on RIVS or Z score for missense from ExAC, its relatively higher haploinsufficient probability^26^ indicates its functional importance (Supplemental Table 2). In addition to these above mentioned deleterious mutations, a single nucleotide substitution (c.454C > A) on the *SNX9* gene (MIM 605952) was observed in a different proband, which result in an amino acid change (p.Q152K) (Supplementary Figure 1 and Table 1). However, this mutation was considered to be tolerable or neutral based on the predictions of the SIFT and LRT software tools, therefore, it was not considered as a possible cause.

Taken together, two *de novo* mutations in *DYNC1H1* and *RTP1* genes identified from two probands may result in severe disruption of protein function.

### Expression profile of *DYNC1H1* and *RTP1* in the developmental human brain

**Figure 2:**
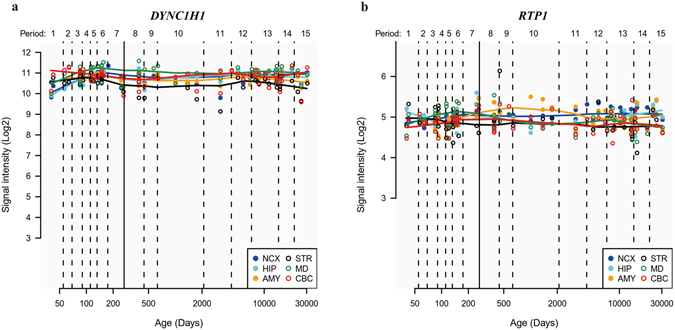
Expression analysis of *DYNC1H1* and *RTP1* in six human brain regions. Expression profiles are shown for *DYNC1H1* (**a**) and *RTP1* (**b**). The expression levels of *DYNC1H1* and *RTP1* are shown for the developmental stages from the embryonic development stage at 50 days to late adulthood (e.g., 30,000 days). A solid line between periods 7 and 8 separates prenatal periods from postnatal periods. CBC, cerebellar cortex; MD, mediodorsal nucleus of the thalamus; STR, striatum; AMY, amygdala; HIP, hippocampus; NCX, neocortex.

To investigate the potential contribution of *DYNC1H1* and *RTP1* genes to human brain development and functions, we firstly assessed the expression profiles of the two genes for different brain regions and developmental stages. Gene expression levels measured using micro-arrays were obtained from the Human Brain Transcriptome database (HBT, http://hbatlas.org/) for 6 human brain regions, including the cerebellar cortex (CBC), mediodorsal nucleus of the thalamus (MD), striatum (STR), amygdala (AMY), hippocampus (HIP) and neocortex (NCX), and 15 human developmental periods, from early embryonic stages to late adulthood. Both *DYNC1H1* and *RTP1* were stably expressed at high levels in all 6 brain regions analyzed throughout the human lifespan (Fig. 2). In addition, *DYNC1H1* expression was approximately two-fold higher than that of *RTP1*. Further analysis of the expression data for the two genes in 11 areas of the neocortex region based on the HBT database revealed similar expression patterns in 6 different brain regions (Supplementary Figure 2). Therefore, we concluded that *DYNC1H1* and *RTP1* may be important for early brain development and normal brain functions considering their stable high expression levels, and are potential candidate genes for brain function related disorders.

### Co-expression and genetic interaction network analyses of *DYNC1H1* and *RTP1*

**Figure 3:**
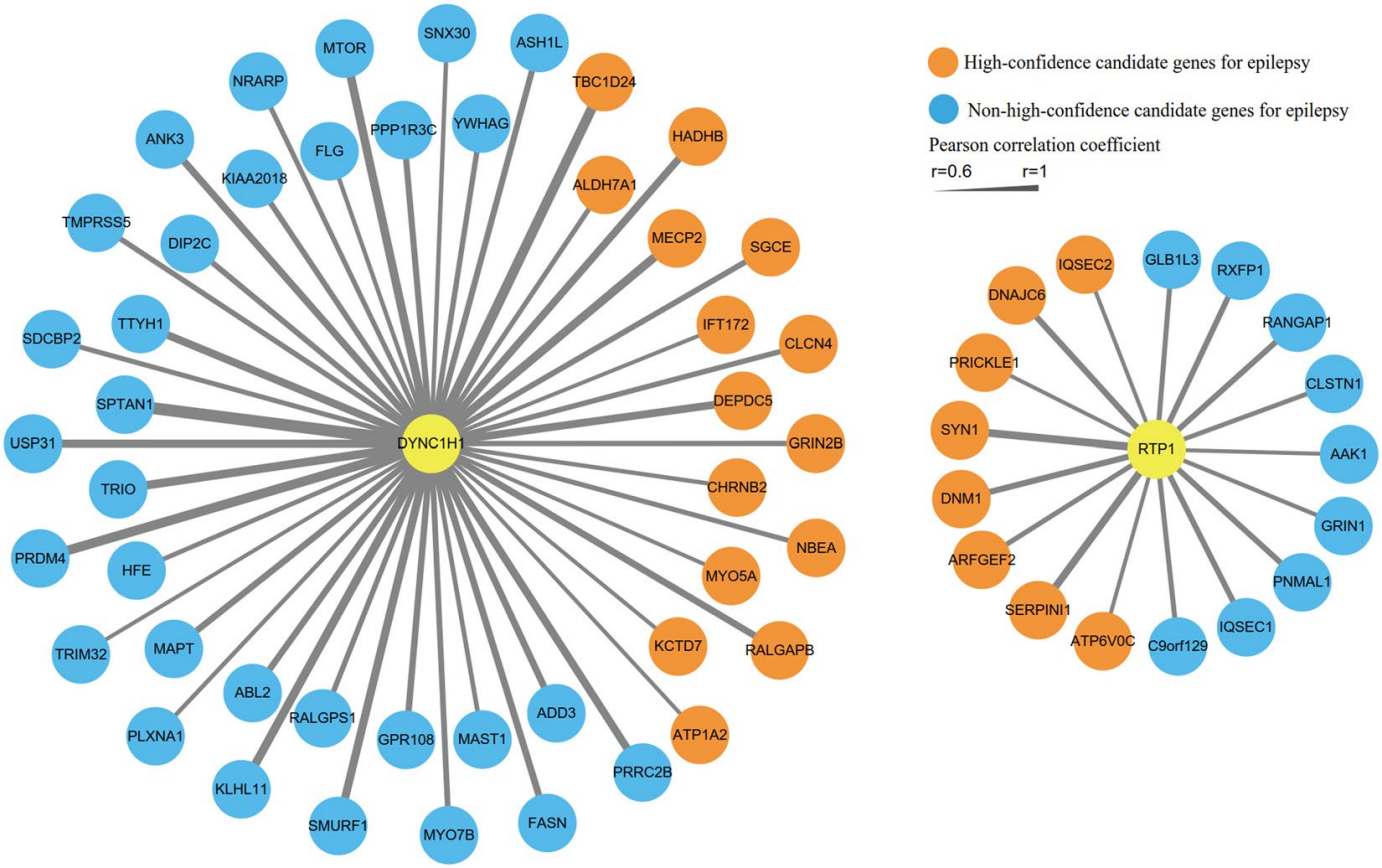
Co-expression network analysis of *DYNC1H1* and *RTP1*. Gene co-expression levels were estimated using the Pearson correlation coefficients (r) between each pair of genes. Candidate epilepsy genes were extracted from the EpilepsyGene database.

To further explore the relationship of *DYNC1H1* and *RTP1* expression with other genes related to epilepsy, we performed a co-expression network analysis based on gene expression data for the human brain throughout development from the BrainSpan database (http://www.brainspan.org/). An obvious co-expression relationship was observed between each of the two genes and epilepsy-associated genes obtained from the EpilepsyGene database (Fig. 3). *DYNC1H1* was co-expressed (Pearson correlation coefficients ≥0.6) with 46 associated epilepsy genes among all co-expressed genes (significance p = 6.6 × 10^−4^, Fisher’s exact test), and in addition, 15 genes (significance p = 0.024, Fisher’s exact test) were considered high-confidence genes based on their relevance to epilepsy according to gene prioritization in the EpilepsyGene database (Fig. 3). In addition, we detected a co-expression relationship between *RTP1* and 17 genes associated with epilepsy (significance p = 0.025, Fisher’s exact test), almost half of which belong to the high-confidence genes (significance p = 0.012, Fisher’s exact test).

**Figure 4:**
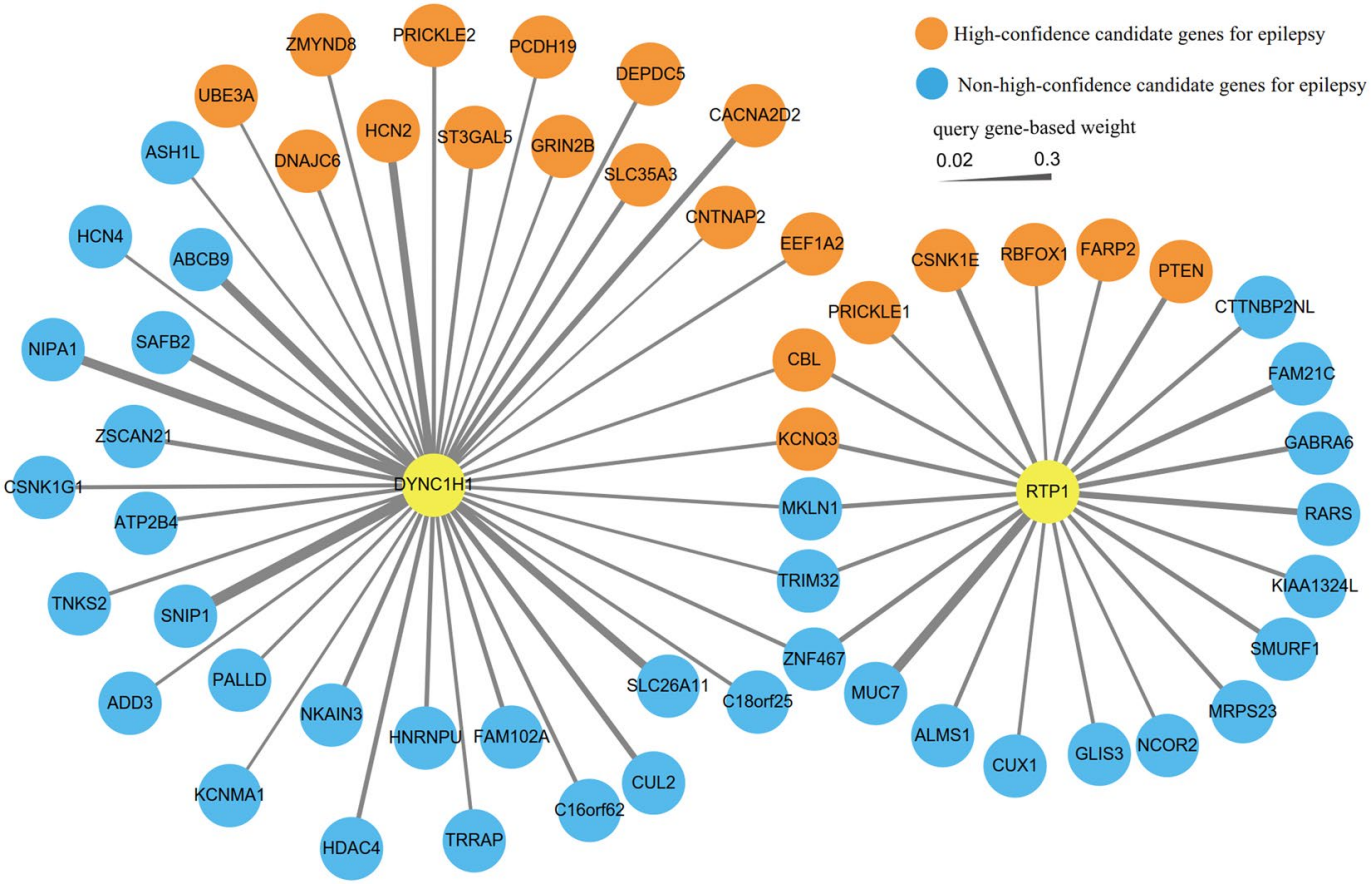
Genetic interaction network analysis of *DYNC1H1* and *RTP1*. A node represents genes, and an edge represents interactions between each pair of genes. Candidate epilepsy genes were extracted from the EpilepsyGene database.

Furthermore, to investigate the functional association between the two genes and other genes associated with epilepsy, we implemented a genetic interactions network analysis based on the interaction dataset collected from the GeneMANIA database. The results revealed that *DYNC1H1* was associated with 41 epilepsy-associated genes obtained from the EpilepsyGene database to different extents (Fig. 4). Similarly, *RTP1* displayed genetic interactions with 22 genes. Within this genetic interaction network, 12 high-confidence genes associated with epilepsy were detected in the network analysis of *DYNC1H1*, in addition to 9 genes for *RTP1* (Fig. 4). Furthermore, five shared genes existed between the genetic interaction networks for *DYNC1H1* and *RTP1*. In particular, *CBL* and *KCNQ3*, two shared genes, were considered high-confidence genes associated with epilepsy (Fig. 4). Overall, the co-expression and genetic interactions network analyses indicated that *DYNC1H1* and *RTP1* may share a similar role with several candidate epilepsy genes.

## Discussion

The *DYNC1H1* gene is located in chromosome 14q32 and was previously implicated in both central and peripheral neuronal dysfunction^27^, associated with syndromes involving spinal nerve degeneration and brain developmental problems^28^. In a previous study, frequent recurrence of mutations in *DYNC1H1* (8 different *de novo* mutations in 16 unrelated probands) was described in a cohort of individuals affected by the malformations of cortical development (MCD) disorder using WES^29^. Interestingly, among the probands with *de novo* mutations in the *DYNC1H1* gene, one proband had clinical symptoms of EE (LGS), and 4 additional probands also possessed epileptic features. Another recent study reported a novel *de novo* mutation in *DYNC1H1* presenting with the clinical phenotype of sporadic congenital Spinal Muscular Atrophy-Lower Extremity Dominant (SMA-LED) and EE^30^. In addition, the DYNC1H1 gene tends to be intolerant to functional genetic variation based on RIVS and Z score for missense from ExAC. Furthermore, the detected *de novo* mutation (c.10174A > G, p.M3392V) in DYNC1H1 was predicted as “deleterious” based on multiple bioinformatics algorithms and was not observed in any large control population variation databases, including ExAC, 1000 Genomes, dbSNP 142 and ESP databases. These observations suggested that the *de novo* mutation in *DYNC1H1* may play an important role in EE.

Notably, *de novo* mutations in the *DYNC1H1* are recurrently detected based on WES studies in probands with different sporadic neuropsychiatric disorders, such as EE, ID and ASD^31^. In a recent study, in 2 unrelated patients with severe ID syndromes, 2 different *de novo* mutations were identified in the *DYNC1H1* gene (Fig. 1b and Table 2)^27^. Notably, one 51-year-old patient with severe ID who developed generalized epileptic seizures at the age of 3 years^27^. Moreover, other 6 different nonsynonymous *de novo* mutations were detected in *DYNC1H1* in 6 different trios from 3 different cohorts associated with ASD (Fig. 1b and Table 2)^15, 32, 33^. Indeed, the shared genetic etiology between EE and other neuropsychiatric disorders, including ASD, and ID, has previously been described^34, 35^. These results suggest that the *de novo* mutations of *DYNC1H1* are shared among EE, ASD and ID, which indicate its possible contribution to these different genetic disorders.

**Table 2:** The shared *de novo* mutations of *DYNC1H1* among ASD, ID and EE.

Chr	Mutation	Protein change	Mutation type	Disorder	Method	Reference
Chr14	c.2467G > T	p.Val823Leu	SNV	ASD	WES	Rubeis S *et al.* Nature^33^
Chr14	c.6698C > T	p.Ala2233Val	SNV	ASD	WES	Rubeis S *et al.* Nature^33^
Chr14	c.7718A > G	p.Asp2573Gly	SNV	ASD	WES	Rubeis S *et al.* Nature^33^
Chr14	c.12797A > T	p.Asn4266Ile	SNV	ASD	WES	Rubeis S *et al.* Nature^33^
Chr14	c.12214G > A	p.Gly4072Ser	SNV	ASD	WES	Iossifov I *et al.* Nature^33^
Chr14	c.925C > T	p.Arg309Cys	SNV	ASD	WES	Krumm N *et al.* Nat Genet^32^
Chr14	c.11465A > C	p.His3822Pro	SNV	ID	WES	Willemsen MH *et al.* J Med Genet^27^
Chr14	c.4552C > T	p. Glu1518 Lys	SNV	ID	WES	Willemsen MH *et al.* J Med Genet^27^
Chr14	c.10174A > G	p.Met3392Val	SNV	EE	WES	In this study

According to previous research, most of the *de novo* mutations in *DYNC1H1* were identified in functional domains in ASD, ID and EE^15, 17, 32, 33^, with the exception of one variant in ID (c.11465A > C, p.H3822P) and one variant in ASD (c.12797A > T, p.N4266I). These results indicate that these domains play an important role in the function of *DYNC1H1*. The *de novo* Met3392Val mutation in *DYNC1H1* identified in this study is located in the stalk domain derived from two of the coiled-coil domains and is considered to maintain dynein function and neuronal physiology. Four unrelated patients with MCD disorder carrying *de novo* mutations in the stalk domain and showing obvious clinical symptoms of EE and early onset epilepsy were recently reported^29^. This finding indicated that disruption of the stalk domain might affect cortical development associated with the occurrence of epilepsy. We are unsure about the exact mechanisms for different genetics variants in *DYNC1H1* underlying the different phenotypes as mentioned above. However, location and nature of the different mutations, genetic background, and other complex factors including environmental influence, mutation timing and epigenetic factors could affect the genotype to phenotype relationship^36–39^.

One deleterious *de novo* missense mutation (c.323T > A) in the *RTP1* gene was identified in another proband. *RTP1* encodes a member of the receptor transporter protein family and can specifically induce functional cell surface expression of odorant receptors in human embryonic kidney cells^40^. Thus far, this gene has not been clearly associated with any neurodevelopmental disorder according to our knowledge. Nevertheless, one *de novo* mutation (c.334A > G; p.M112V) in *RTP1* was recently described in an EE proband in a cohort of 264 trios based on WES^9^. Although *RTP1* does not tend to be evolutionarily constrained based on RIVS or Z score for missense variant from ExAC, RTP1 is most likely a haploinsufficient gene. Furthermore, this mutation in *RTP1* (c.323T > A) was not observed in any large control population through searching public variation database including ExAC, ESP and 1000 Genomes. These observations indicated that *RTP1* may be a potential candidate gene for EE.

In recent years, WES has proved to be an efficient diagnostic approach for Mendelian diseases^41^. However, some limitations in WES technique impede high-efficiency detection for INDELs and CNVs, and *de novo* mutations from noncoding regions of UTR, introns, or intergenic regions, and synonymous mutations are usually ignored^42^. In addition, for some GC-rich regions, it is difficult to obtain effective sequencing coverage and depth of sequencing for variant detection^43^. These factors are likely to prevent the detection of possible deleterious mutations. The solution of these problems generally includes algorithm improvement, increase of read depth in target regions and application of whole-genome sequencing (WGS)^42, 43^. Nevertheless, it should be noted that approximately 85% of known disease-causing variants are in the coding regions^44, 45^. WES still represents a powerful tool for diagnosis and is adopted by many studies^41, 42^.

## Methods

### Patient recruitment

We collected samples from 4 unrelated West syndrome trios of Han Chinese ancestry from the Second Affiliated Hospital & Yuying Children’s Hospital of Wenzhou Medical University. This study protocol was approved by the Hospital Ethics Committee, and was carried out in accordance with the approved guidelines. Written informed consent was obtained from all participants or their caregivers prior to peripheral blood and clinical data collection. These cases comprised 3 males and 1 females with obvious clinical symptoms of West syndrome, characterized by refractory seizures and cognitive arrest or regression. All epilepsy diagnoses were evaluated based on an assessment of clinical seizure presentation and EEG recorded by a pediatric neurologist with experience in the clinical diagnosis of epileptic encephalopathies.

### Whole-exome sequencing

Genomic DNA samples were extracted from 1 ml of peripheral blood leukocytes obtained from the parents and probands using the QIAGEN DNeasy Blood & Tissue Kit (Qiagen, Valencia, CA, USA). DNA quality and quantity were assessed using a NanoDrop 2000 spectrophotometer (Thermo Scientific, Wilmington, DE, USA). Approximately 2 µg of high-quality genomic DNA from each sample was prepared as the starting material for generating the sequencing library using the Agilent SureSelect Library Prep Kit according to the manufacturer’s instructions. Through target enrichment of DNA samples and construction of a hybridization library, the whole exome was captured using the Agilent SureSelect Human All Exon v5 Kit (Agilent Technologies, Santa Clara, CA, USA). In addition, all sheared DNA samples were tagged through PCR using an index (barcode) sequence. After the purification and quality assessment of the sample libraries, the samples were pooled according to mass, followed by multiplex sequencing using an Illumina HiSeq2000 sequencer (San Diego, CA, USA.) with 101 bp paired-end reads.

### Data processing and *de novo* mutation detection

The raw sequencing reads generated through WES for the 12 individual samples were processed to remove sequence adapters and low-quality reads using the Trim_Galore program. Only sequencing reads with Phred-scaled quality scores greater than 30 and read lengths greater than 80 bp were retained for further analysis. The sequencing reads were aligned to the human reference genome (GRCH37/hg19) using the BWA program (version 0.7.12). After read mapping, duplicated reads and reads mapped to multiple genome locations were removed with the Picard software tool. GATK tools were used for the read realignment, quality score recalibration and SNP/InDel variant calling. Two software tools (ForestDNM and mirTrios) were used for *de novo* mutation detection, and mutations indicated to be *de novo* using both tools were considered as reliable *de novo* mutations for further analysis in the present study.

### *De novo* mutation annotation and evaluation of the tolerance of genes

All variants were annotated using the ANNOVAR software tool and in-house codes. The consequences of mutations for protein products were inferred based on RefSeq gene annotations (http://www.ncbi.nlm.nih.gov/refseq/). Functional predictions for missense variants were obtained using 4 software tools: SIFT, LRT, SiPhy, and VEST3. A missense variant was considered deleterious based on the concordance of four generic damage prediction tools. For each sequence variant, the allele frequency (AF) was obtained from normal population variant databases, including dbSNP (http://www.ncbi.nlm.nih.gov/projects/SNP/), 1000 Genomes (http://www.1000genomes.org/), ESP6500 (http://evs.gs.washington.edu/EVS/), ExAC (http://exac.broadinstitute.org/), CG69 (http://www.completegenomics.com/public-data/69-genomes/) and GWAS Catalog (https://www.ebi.ac.uk/gwas/). Variants with AF <0.001 according to 1000 Genomes, ESP6500 and ExAC were defined as rare variants.

To assess the intolerance of genes to functional genetic variation, we utilized the predicted haploinsufficient probability^26^, residual variation intolerance score (RVIS) based on the ESP6500^25^ dataset and Z score for missense variants from ExAC^22^.

### Primers and Sanger sequencing validation

To validate each potential *de novo* mutation detected in the present study, 100 ng of genomic DNA from each sample was used to amplify the target mutation region through conventional PCR and Sanger sequencing. All primers in these assays are listed in Supplemental Table 3.

### Gene co-expression and genetic interaction network

Gene expression data were obtained from the BrainSpan (http://www.brainspan.org/) database, which shows gene expression levels for specific brain regions obtained through RNA sequencing and exon microarray techniques. Gene co-expression levels were estimated based on the Pearson correlation coefficients (r) between each pair of genes. We extracted each pair of genes exhibiting high co-expression correlation coefficient (absolute r ≥ 0.6) compared with target genes and candidate epilepsy genes in the EpilepsyGene database^46^. Enrichment analysis of co-expressed epilepsy genes by using one-sided Fisher’s Exact test. To generate a human genetic interaction network, the direct protein-protein interaction dataset was collected from GeneMANIA^47^, based on the InWeb database or the Cytoscape program plugin^48^. In addition, GeneMANIA was employed to calculate the number of nodes and edges between the target genes and candidate epilepsy genes from the EpilepsyGene database. In the genetic interaction network, a node represents genes, and an edge represents interactions between two genes. The thickness of the edge represents the degree of genetic interaction. Both gene co-expression and the genetic interaction network were visualized using the Cytoscape program.

## Electronic supplementary material

Supplementary Information
