# Supplementary material for: Whole-exome sequencing identifies a novel de novo mutation in DYNC1H1 in epileptic encephalopathies
Source: Sci Rep. 2017 Mar 21;7:258. doi: 10.1038/s41598-017-00208-6 (PMC5428060; doi:10.1038/s41598-017-00208-6)
Supplement: Supplementary file 1 — Supplementary Information [file 41598_2017_208_MOESM1_ESM.pdf]

Whole-exome sequencing identifies a novel *de novo* mutation in *DYNC1H1* in epileptic encephalopathies

Zhongdong Lin[1,3,†], Zhenwei Liu[2,†], Xiucui Li[3], Feng Li[3], Ying Hu[3], Bingyu Chen[4], Zhen Wang[4,*], Yong Liu[1,*]

[1]Institute of Neurobiology, Xi'an Jiaotong University Health Science Center, Xian, China

[2]Institute of Genomic Medicine, Wenzhou Medical University, Wenzhou, China

[3]Department of Pediatric Neurology, The Second Affiliated Hospital and Yuying Children's Hospital, Wenzhou Medical University, Wenzhou, China

[4]Research Center of Blood Transfusion Medicine, Key Laboratory of Laboratory Medicine, Ministry of Education, Zhejiang Provincial People's Hospital, Hangzhou, China

[†]These authors contributed equally to this work

*Correspondence should be addressed to Zhen Wang (yuchen6046@163.com) and Yong Liu (liuy5599@mail.xjtu.edu.cn)

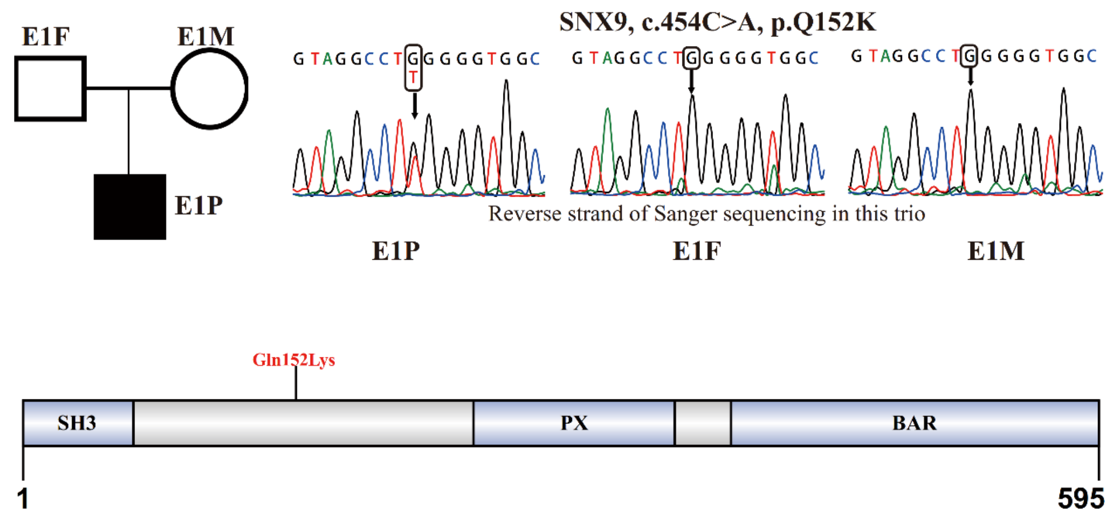

Supplementary figure 1. ***De novo*** **mutations identified in** ***SNX9*****.** The *SNX9* variant (c.454C>A, p.Q152K) of trio E1 were confirmed using Sanger sequencing and Schematic representation of the SNX9 protein

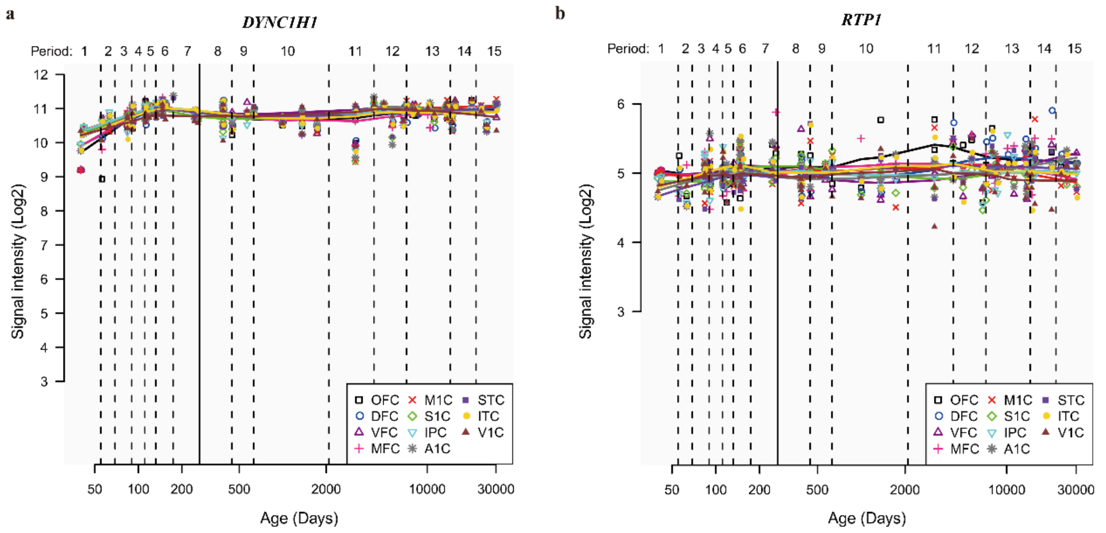

Supplementary figure 2. Expression analysis of *DYNC1H1* and *RTP1* in 11 areas of the neocortex (NCX). OFC, orbital prefrontal cortex; DFC, dorsolateral prefrontal cortex; VFC, ventrolateral prefrontal cortex; MFC, medial prefrontal cortex; M1C, primary motor cortex; S1C, primary somatosensory cortex; IPC, posterior inferior parietal cortex; A1C, primary auditory cortex; STC, posterior superior temporal cortex; ITC, inferior temporal cortex; V1C, primary visual cortex.

Supplemental Table 1. Summary of whole-exome sequencing data

Sample	Clean data (Gb)	Initial bases on target (Mb)	Alignment rate (%)	Base covered on target(Mb)	Fraction of effective bases on target (%)	Average sequencing depth	4× coverage (%)	10× coverage (%)	20× coverage (%)
E1F	3.18	50.34	99.48	49.06	56.60	61.38	96.00	92.40	83.90
E1M	4.24	50.34	99.61	49.07	57.20	84.35	96.40	94.00	88.70
E1P	3.09	50.34	99.51	49.03	57.40	61.77	95.90	92.10	83.50
E2F	2.12	50.34	99.48	48.69	56.20	41.01	93.60	86.00	71.30
E2M	3.72	50.34	99.45	48.68	55.30	63.43	95.20	91.10	82.70
E2P	3.47	50.34	99.49	49.19	55.70	66.51	96.20	93.00	85.30
E3F	3.92	50.34	99.55	48.98	58.00	77.36	95.90	92.70	86.10
E3M	3.43	50.34	99.52	48.94	56.20	65.95	95.70	92.20	84.30
E3P	4.72	50.34	99.58	48.99	56.70	86.45	96.10	93.30	87.80
E4F	3.79	50.34	99.51	48.98	55.90	69.27	96.00	92.70	85.50
E4M	4.30	50.34	99.43	48.77	55.70	72.83	95.60	92.40	85.60
E4P	3.76	50.34	99.42	48.66	55.70	63.76	95.20	91.20	83.00
**Mean**	3.78	50.34	99.51	48.93	56.38	70.25	95.72	92.15	84.56
**SD**	0.67	0.00	0.06	0.18	0.79	12.33	0.72	1.93	4.19

Supplemental Table 2. The evaluation of the tolerance of genes with de novo mutation in this study

Trio	Gene	Probability of HI	RVIS	RVIS Percentile	Z score for missense from ExAC
**E1**	*SNX9*	0.143	-0.13	43.91%	1.31
**E2**	*RTP1*	0.736	0.06	58.84%	0.76
**E3**	*DYNC1H1*	0.436	-6.01	4.13%	13.88

Not: HI, haploinsufficiency; RVIS, residual variation intolerance score; Lower RVIS percentiles and higher Z scores correspond to higher constraint

Supplemental Table 3. PCR information for the validation of de novo mutation in this study

Trio	Gene	Mutation	Primers (5'→ 3')	Length(bp)
**E1**	*SNX9*	c.454C >A, p.Q152K	Forword: CAACTGGGACACTGCCTTCG<br>Reverse: CTTCCCTGTCTCTCCCACTCAG	110
**E2**	*RTP1*	c.323T>A, p.I108N	Forword: AGTGCACCAAGCGCATTTT<br>Reverse: GGCCCAACCACGGAATCTTA	858
**E3**	*DYNC1H1*	c.10174A>G, p.M3392V	Forword: TCAGTCACTGGGGCAATGAG<br>Reverse: AAGAGGTCACCTTTCACACGG	279
